# Pollination of *Ficus elastica*: India rubber re-establishes sexual reproduction in Singapore

**DOI:** 10.1038/s41598-017-09873-z

**Published:** 2017-09-14

**Authors:** Rhett D. Harrison, Kwek Yan Chong, Nguyet Minh Pham, Alex T. K. Yee, Chow Khoon Yeo, Hugh T. W. Tan, Jean-Yves Rasplus

**Affiliations:** 1 World Agroforest Centre (ICRAF), East & Southern Africa Regional Office, 13 Elm Road, Woodlands, Lusaka, Zambia; 2 Department of Biological Sciences, National University of Singapore, 14 Science Drive 4, Singapore, 117543 Republic of Singapore; 3 INRA, UMR 1062, 34988 Montferrier-sur-Lez cedex, France

## Abstract

*Ficus elastica*, otherwise known as India Rubber (although its geographical origins are unclear), was an important source of latex in the early 19^th^ century and was widely cultivated in tropical Asia. Like all figs, *F. elastica* is dependent on tiny, highly specific wasps for pollination, and detailed studies based out of Singapore in the 1930s suggested that through the loss of its pollinator *F*. *elastica* was extinct in the wild. However, around 2005 wild seedlings of *F*. *elastica* began appearing in Singapore. We identified the pollinator as *Platyscapa clavigera*, which was originally described from *F*. *elastica* in Bogor in 1885. A visit to Bogor Botanical Gardens revealed that not only was *F. elastica* being pollinated by *P. clavigera* in the gardens, but there was clear evidence it had been reproducing naturally there over many decades. Although Singapore has a native fig flora of over 50 species, *F*. *elastica* went unpollinated for at least 70 years and probably from the time it was introduced during the 19^th^ century. These observations illustrate the extraordinary specificity of this interaction and, through the fig’s ability to wait for its pollinators, demonstrates one way in which such highly specific interactions can be evolutionarily stable.

## Introduction

Before the discovery of vulcanisation in 1844, latex from a wide range of tree species was harvested, including the India Rubber tree, *Ficus elastica* ^1–3^. In the late 18^th^ and 19^th^ centuries increasing demand for latex driven by the industrial revolution in Europe led to widespread cultivation of *F. elastica* in tropical Asia^2, 3^. However, with the discovery of vulcanisation the Para rubber tree (*Hevea brasiliensis*), which is from the Amazon, became a superior source of latex and the India Rubber plantations were eventually abandoned^1^. Unfortunately, this early widespread cultivation of India Rubber obscured its geographical origins^4^. In 1887, King described it as a wet forest species from the Himalayan region of NE India through Malaya^5^. However, E.J.H Corner, working out of the Singapore Botanic Gardens in the 1930s and a renowned regional expert on *Ficus* taxonomy and biogeography, noted that it did not occur naturally in the lowland forests of Malaysia or Thailand^4^. Moreover, he pointed out that early descriptions of *F. elastica* as being abundant in the forests of Assam and Burma were not corroborated by more recent surveys^4^, suggesting the earlier observations may have been of abandoned plantations. In 1874, Strettell found large *F. elastica* individuals and epiphytic seedlings in limestone forest in north Burma^2^ and the renowned Dutch botanist Blume described it from limestone forest in west Java^4^. Corner also noted other collections from limestone outcrops in Sumatra and Peninsular Malaysia^4^. In addition, widespread cultivation of *F. elastica* from cuttings led to the emergence of sterile clones, which even today constitute the majority of individuals in cultivation. Some cultivated individuals are fertile, but from India to Singapore these were never know to set seed. Hence, Corner suggested that *F. elastica* was extinct in the wild^4, 6^.

*Ficus elastica* is a familiar plant through its cultivation as both an indoor and outdoor ornamental. Throughout the tropics it is a common tree in streets and parks and as an indoor ornamental it qualifies as one of the most widely distributed plants today. *Ficus elastica* belongs to the fig genus, which has a roughly pan-tropical distribution with approximately 750 species globally^7, 8^. As with other members of the Mulberry family (Moraceae), all figs have latex. Although the latex of *F. elastica* is particularly copious and viscous, which presumably led to its cultivation for latex production. Figs have a unique pollination system, which is regarded as a classic example of an obligate mutualism^8, 9^. The fig fruit – strictly speaking an inflorescence – is an urn-shaped structure lined with the fig’s tiny flowers and closed at the neck by overlapping bracts. When the fig is receptive, the bracts loosen enabling the female fig wasps to enter, although they lose their antennae and wings in the process. Once inside, the wasps pollinate the uniovulate female flowers and lay eggs in some of them. An ovule that receives a wasp egg develops into a gall, which protects and nourishes the larva within. Ovules of pollinated flowers that do not receive a wasp egg develop into seeds in the normal way. The system is an example of seed predator pollination, whereby the plant gives up a proportion of its seeds to feed developing pollinator larvae^10^. Approximately one month later, the adult male wasps chew their way out of their galls and mate with the gall-enclosed females. The females then emerge, collecting pollen on the way, and disperse in search of a receptive fig of the same species in which to lay their eggs. The female wasps use wind assisted dispersal^11–13^, sometimes traveling huge distances^14^, before locating a tree with receptive figs of the correct species by identifying the specific floral bouquet^15^. The fig plant is, therefore, entirely dependent on the fig pollinator for pollination services and the pollinator is entirely dependent on the fig plant for rearing its offspring. Moreover, it is a highly specific interaction. In any particular area a majority of fig species are pollinated by a single, species-specific pollinator – although examples of geographical turn over in pollinator species^16–18^, sympatric pollination by two or more pollinator species^19, 20^ and the existence of cheaters (co-“pollinating” fig wasps that do not pollinate)^21^ have been described. The genus is estimated to be slightly over 75 million years old and phylogenetic studies indicate a general (but imperfect) pattern of co-speciation down the fig and pollinator lineages^22^. As mentioned above, because *F. elastica* was never observed to produce seeds (and wild seedlings were also never observed), Corner assumed that the pollinator must be extinct and that, therefore, *F*. *elastica* was extinct in the wild^6^.

Around 2005 in Singapore, young plants of *F*. *elastica* began appearing, including some in odd places such as on top of a bus shelter, indicating a production of wild seedlings. A quick investigation revealed the presence of seeds inside the figs of *F. elastica*. Given our understanding of fig biology, there were three possible explanations. (1) The wild seedlings of *F. elastica* were clones produced by apomixis. (2) The pollinator of a sympatric fig species had colonised the *F. elastica* population and now served as its pollinator. (3) The original pollinator of *F*. *elastica* was in fact not extinct, but had only recently colonised Singapore. We set out to examine these three hypotheses and, in the process, shed some light on the biology of *F*. *elastica*.

## Results

Microsatellite analyses demonstrated that wild seedlings of *F. elastica* in Singapore were not clones (Figs S1 and S2). Seedlings inherited some alleles from a nearby adult, presumably the maternal parent, but other alleles came from other non-identified individuals. This demonstrated that *F*. *elastica* is breeding in Singapore, and hence we could reject our first hypothesis.

**Figure 1:**
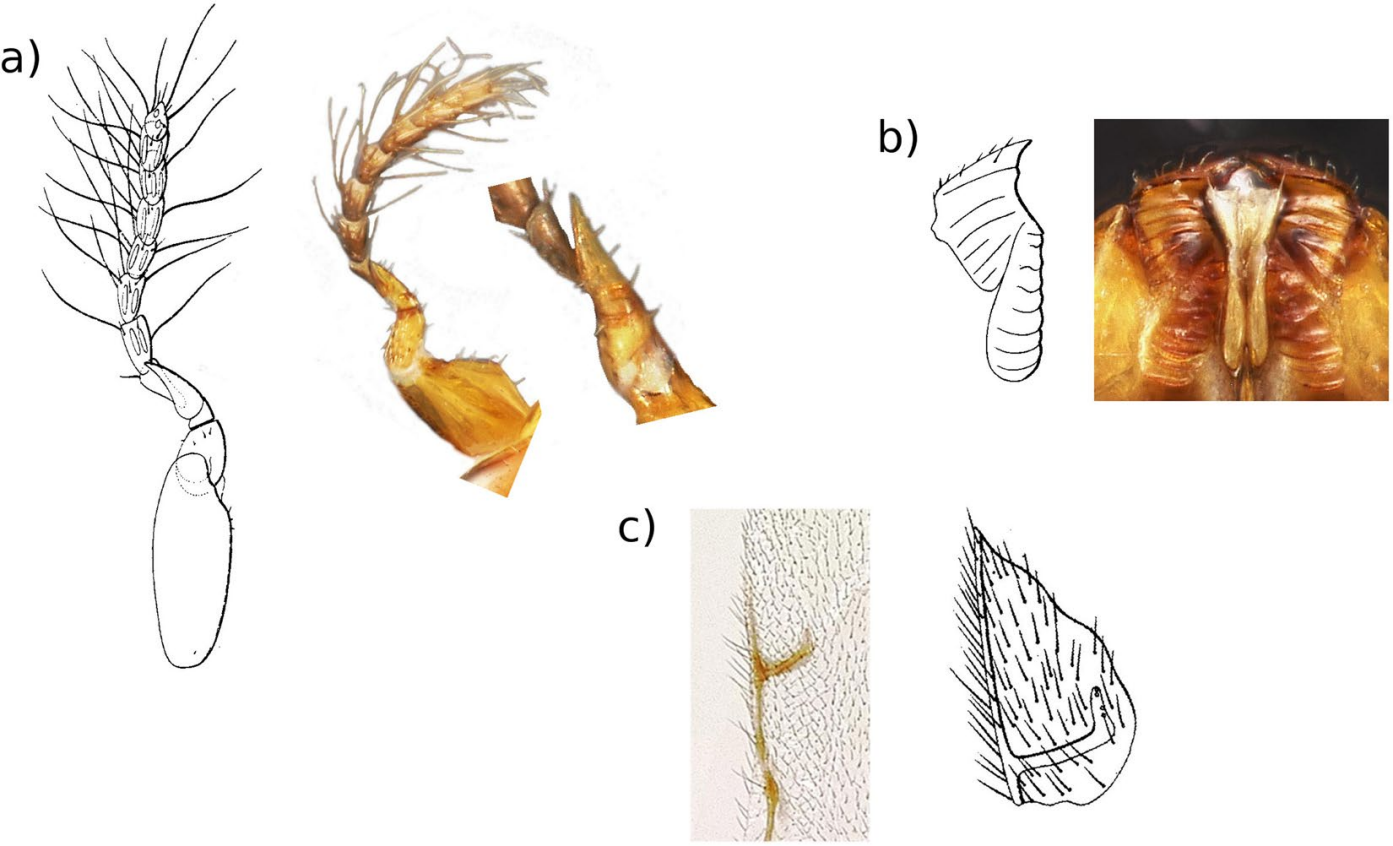
Details of diagnostic characters for female fig wasps. Sketches are from the original description of *Platyscapa clavigera* ^24^. Images are from wasps collected from *F. elastica* in Singapore. (**a**) Antennae (with enlarge inset of antennal spike). (**b**) Mandibular appendage. (**c**) Stigmal vein. Images were produced with an EntoVision Premium Portable Imaging System, comprising a Leica M16 zoom lens, a JVC KY-75U 3CCD digital camera and a portable computer workstation running EntoVision Imaging Suite software (GT Vision, Hagerstown, MD U.S.A.).

**Figure 2:**
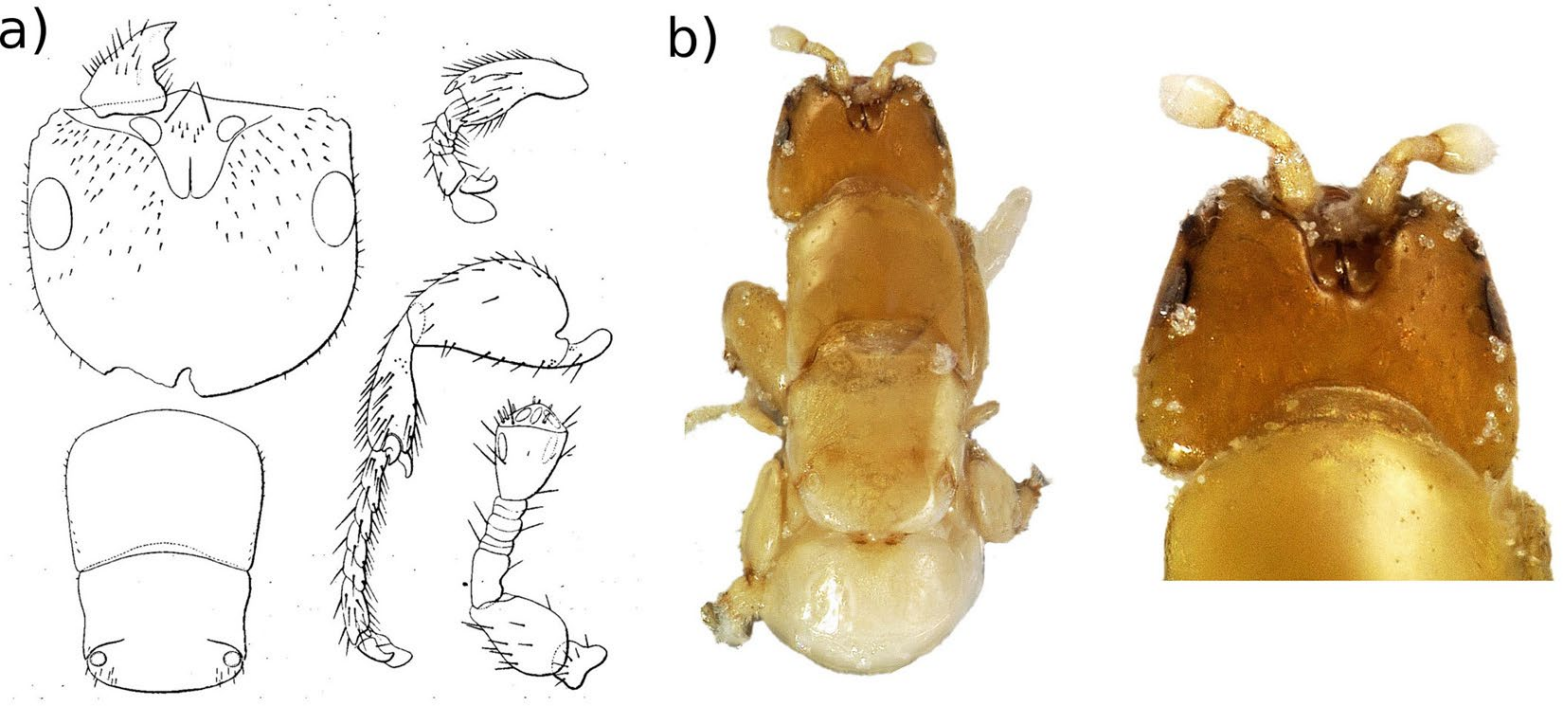
Details of diagnostic characters for male fig wasps. (**a**) Sketches are from the original description of *Platyscapa clavigera* ^24^. (**b**) Wasps collected from *F. elastica* in Singapore. Images were produced with an EntoVision Premium Portable Imaging System, comprising a Leica M16 zoom lens, a JVC KY-75U 3CCD digital camera and a portable computer workstation running EntoVision Imaging Suite software (GT Vision, Hagerstown, MD U.S.A.).

To decide between the second and third hypotheses, we collected emerging fig wasps and conducted a morphological examination of the pollinator. A pollinator was described from *F. elastica* in Bogor in 1885^23, 24^. Unfortunately, the type material was lost and hence we had to rely on the description, including drawings. Nevertheless, a comparison between the wasps collected by us in Singapore and the description shows they are remarkably similar (Figs 1 and 2). Key characters for identifying female fig pollinators include the antennae (Fig. 1a), the mandibular appendage (a teeth-like structure attached to the underside of the mandibles, which helps the wasps pull themselves through the bracts when entering the fig) (Fig. 1b) and the shape of the stigmal vein on the fore wings (Fig. 1c). Male fig pollinators have very reduced traits making them difficult to identify. However, again in the shape and relative size of the antennae, the dorsal view of the head and the dorsal view of the mesosoma (Fig. 2b), the pollinators collected from Singapore are similar to the 1885 description of the pollinator of *F. elastica* from Bogor. Hence, we tentatively identified the pollinators collected in Singapore as *Platyscapa clavigera* (Mayr 1885) (it was originally named as *Blastophaga clavigera*)^24^.

**Figure 3:**
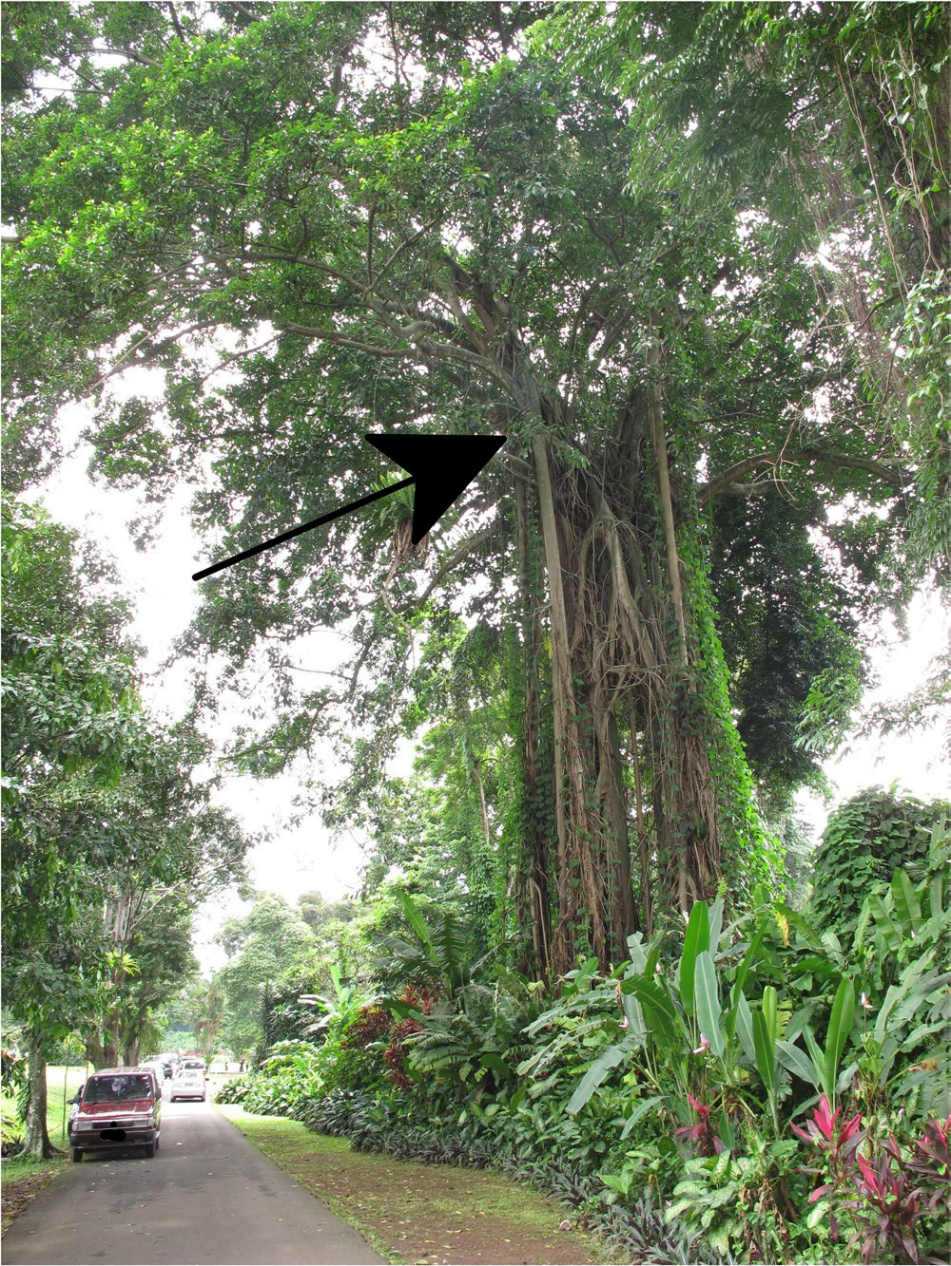
Image of a *F. elastica* hemi-epiphyte in the Kebun Raya Bogor (Bogor Botanical Garden). The arrow shows the point at which the aerial roots connect with the short truck and branches, and indicates the point of attachment of the original epiphytic seedling.

**Figure 4:**
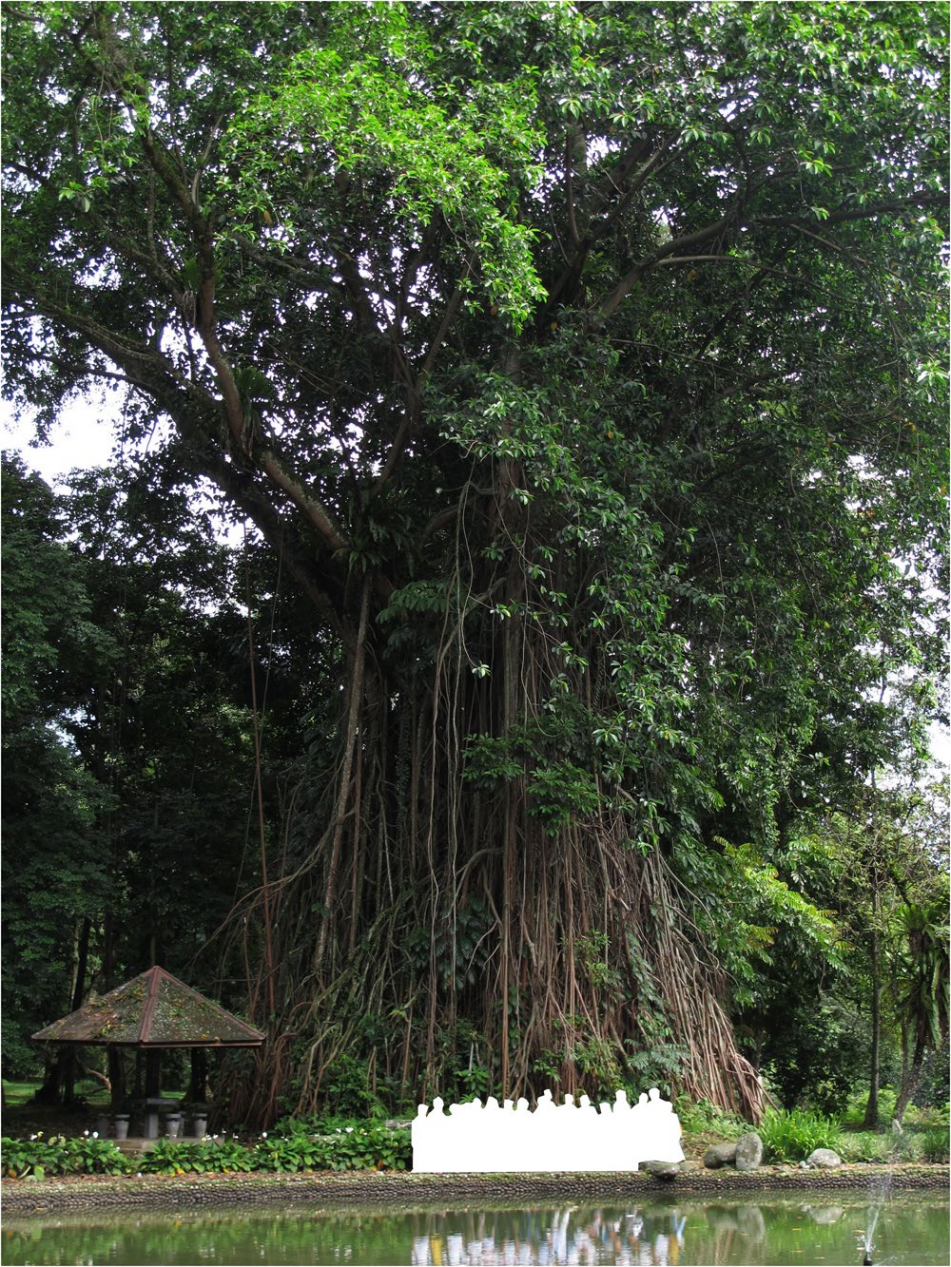
Image of a large *F*. *elastica* individual from Kebun Raya Bogor (Bogor Botanical Garden).

To check on this identification we visited Kebun Raya Bogor (Bogor Botanic Gardens) in 2012 and inspected the *F*. *elastica* growing there. We found several individuals bearing pollinated figs and inspection of the pollinating wasps found them to be the same species as those collected from Singapore. Moreover, the presence of several large stranglers indicated that *F*. *elastica* had been reproducing naturally in the gardens over many years (Figs 3 and 4). Stranglers are hemi-epiphytes, meaning they begin life as an epiphytic seedling in the fork of a large tree and then send roots down the host truck to connect with the ground^25^. In mature individuals it is possible to deduce the position on the host tree of the original epiphytic seedling, as this is the point at which the aerial roots connect to the trunk and branches (Fig. 3). Several of the *F*. *elastica* in the Kebun Raya Bogor had aerial roots extending over 15 m up the trunk of their host, making it inconceivable that someone had planted them. Moreover, the size of some individuals (Fig. 4) suggested they were several decades, possibly a century or more, in age.

Although varying according to the species, figs often harbour a diverse fauna of non-pollinating fig wasps. However, we did not collect or observe any other species of fig wasp on *F*. *elastica* in either Singapore or Bogor.

## Discussion

We conclude that the pollinators of *F. elastica* collected in Singapore are *P*. *clavigera* ^23, 24^. Moreover, based on our observations of large stranglers in the Kebun Raya Bogor we suggest that *P. clavigera* has persisted in Bogor for a long time. Since making these observations we discovered recent reports of natural reproduction in *F. elastica* in Ujong Kulong in the extreme west of Java^26^ and Thung Yai Naresuan East Wildlife Sanctuary in Umphang district, Thailand^27^. However, neither report established the identity of the pollinators. Both these sites have calcareous soils lending support to the suggestion that *F. elastica* is a limestone specialist in its natural condition. As mentioned earlier, in 1874 Strettell also found both large individuals and epiphytic seedlings of *Ficus elastica* in a limestone forest in north Burma, whereas elsewhere he reports finding only planted individuals^2^. We have inspected dozens of *F. elastica* individuals bearing figs in urban areas in Peninsular Malaysia, Thailand and Yunnan but we have yet to observe any that have been pollinated. Hence, we suggest that, in line with Corner’s suggestions^4^, *F*. *elastica* probably originated as a limestone specialist and spread to other areas through cultivation. If we assume this to be correct, its known natural distribution includes western Java, southern Sumatra, Peninsular Malaysia, western Thailand and north Burma^2, 4, 26, 27^. The alternative hypothesis that *P. clavigera* is a lowland moist forest species that became extinct throughout a native range centered on NE India and Burma^5^ but persisted in Bogor and isolated limestone areas seems much less likely. Although local, temporary extinctions of some fig pollinators have been recorded in extreme climatic events^28^, these did not involve *Platyscapa* and related genera, which are capable of long distance wind-assisted dispersal^11–14^, and over the long-term the fig – fig pollinator interaction appears very robust to such stresses^22^. Moreover, although reduction in populations of fig hosts and fragmentation of their habitat through deforestation is a potential threat to the survival of fig species^29^, and there are preliminary indications of pollination failure among some species in fragmented forests^30^, the cultivation of *F. elastica* in plantations would have created a huge, widespread population of hosts, even considering that a large proportion were probably sterile.

These findings illuminate a number of interesting aspects to the ecology of the fig – fig pollinator interaction. Fig wasps are known to use wind assisted dispersal and through genetic matching have been shown to travel in excess of 150 km^14^. They have also been recorded several tens of kilometers offshore at ships lights and have been quick to colonise exploded volcanic islands^31, 32^, indicating that sea crossings would not appear to be a barrier to dispersal. Hence, it seems surprising that *P. clavigera* was present in the region, but failed to reach Singapore until 2005. It is around 70 years since Corner’s observations, but it seems likely that the pollinator has been absent from the time *F. elastica* was first introduced to the island during the 19^th^ century. Nevertheless, to reach Singapore *P*. *clavigera* would have had to disperse across extensive lowland moist forests that harbour few or no *F*. *elastica*. It is also quite possible that habitat specialisation presented a barrier to successful colonisation of Singapore by *P*. *clavigera* until recently. Throughout a large part of East Africa, *Ficus sur* is pollinated by two wasp species, one of which occurs in forested areas and one in savannas^33^.

The fact that *F. elastica* was present in Singapore but went unpollinated for at least 70 years but probably over 150 years, also illustrates the extreme specificity of the interaction. There are about 50 native species of fig in Singapore^8^, including several species pollinated by *Platyscapa* and related genera. Elsewhere, it has been found that in the absence of the legitimate pollinator a low proportion of figs may be entered by the pollinators of other species, indicating a breakdown in specificity^30, 34^. Fig species with multiple sympatric pollinators have also been discovered^19, 20^. However, the specificity of the *F. elastica* - *P*. *clavigera* interaction was maintained over many decades, despite ample opportunity for breakdown. It would be interesting to investigate the degree of specificity across a large sample of fig - fig pollinator interactions and examine the ecological and evolutionary correlates. Natural populations of *F. elastica* and *P*. *clavigera* would also appear to be isolated in widely separate patches of limestone forest and therefore would make an interesting topic for a population genetic study^18^.

Last, the *F*. *elastica* - *P*. *clavigera* story illustrates how such extremely specialised interactions can also be evolutionarily stable. Although *F*. *elastica* in Singapore were cultivated, individual trees can live at least a century and possibly much longer. The growth rates of large stranglers are not well understood^25, 35^, but it seems unlikely that an individual as colossal in size as the one by the pond in the Kebun Raya Bogor (Fig. 4) could be anything less than several centuries old (the oldest fig of known planting date is a Bodhi tree (*F*. *religiosa*) which was planted in Sri Lanka in 255 BC)^36^. Although almost certainly reaching Singapore by artificial means, *F*. *elastica* has been able to consolidate this range extension by waiting for its pollinator to catch up. There are also some examples of other fig species that have been transported around the world via the horticultural trade, yet are pollinated by the ‘correct’ fig wasp species many thousands of miles outside their natural range^37–39^. In these cases, presumably the pollinators were also transported via the horticultural trade, as larvae in developing figs. The ability of figs to wait for their specific pollinator to catch up over decades, and possibly centuries, has likely contributed to the stability of the interaction over evolutionary time-scales as different lineages dispersed around the globe^22^. Thus, after an extended period of abstinence, India Rubber has rediscovered sex in Singapore.

## Methods

### Microsatellite analyses

The leaves of *F*. *elastica* were collected on 8 June 2011 from two large adult trees and two spontaneously growing seedlings found in the vicinity of the Chinese Heritage Centre (1.343820°N, 103.684023°E) of at the Nanyang Technological University campus, Singapore. Leaf samples were surface-sterilized with 30% bleach then autoclaved milliQ water for 10 minutes. The sterilized leaf samples were then stored in a −80 °C freezer on the same day.

Prior to DNA extraction, the four leaf samples of *F. elastica* were cut into pieces. Then, 0.5 g of the cut leaves of each sample was ground with liquid nitrogen using a mortar and pestle. The frozen and ground leaf tissue was subsequently used for DNA extraction, following standard protocols^40, 41^ with minor modifications. We added 600 μL of extraction buffer to the ground tissue and incubated for 60 °C for 25 min. We then added 720 μL of chloroform-octanol and inverted the tubes 20 times. The emulsion was spun at 13,000 rpm for 15 min, and then 400 μL of the aqueous phase was transferred to a new 1.5 mL tube. To this we added 200 μL of 5 M NaCl, 800 μL of −20 °C 95% ethanol, and placed the tube in ice for 1 h. The tube was then spun at 13,000 rpm for 10 min and the supernatant was poured out. The remaining pellet was washed with 50 μL of 0 °C ethanol and spun again at 13,000 rpm for 10 min. The ethanol was removed and the pellet dried by leaving it uncovered for 20 min at 37 °C. Finally, the pellet was dissolved with 30 μL of milliQ water. Eight microsatellite primer pairs developed from *Ficus carica* ^42^ were used to amplify DNA extracted from these leaves. The Polymerase Chain Reaction (PCR) amplification protocol also followed that of 34, performed in a final volume of 50 μL, comprising 27.5 μL of water, 5 μL of 10 × PCR buffer, 5 μL of 2 mM dNTP mix, 1 μL of the reverse primer, 1 μL of the forward primer, 6 μL of 2 mM MgCl2, 0.5 μL of Taq polymerase, and 4 μL of DNA. The following PCR cycles were used: 1 cycle of 94 °C for 4 min; 35 cycles of 94 °C for 30 s, 50–55 °C for 45 s and 72 °C for 1 min; and 1 final extension cycle of 72 °C for 4 min. The PCR products were denatured by incubation at 95 °C for 5 min and placed on ice. Agarose (3%) in 1 × TBE with 12 μL of SYBR Safe was prepared for the gel electrophoresis. At 70 V, 25 μL of PCR products were mixed with 6 × loading dye and loaded in each well. The ladders used were: Fermentas GeneRuler 1 kb DNA Ladder, Fermentas GeneRuler 100 bp DNA Ladder, and Fermentas O’RangeRuler 20 bp DNA Ladder.

### Collection of fig wasps

Mature figs of *F. elastica* were placed in sealed plastic bags and left indoors at room temperature until the female wasps emerged naturally. All fig wasps observed in the bags within 24 h were collected. Emerged wasps were placed directly into vials with 80% ethanol. To collect the male wasps we then opened the figs and searched among the galls. We collected wasps from 8 trees between 10 Oct 2009 and 21 May 2010.

### Fig wasp morphological observations

To produce high quality images, some specimens were point-mounted on grey card in order to avoid loss of contrast caused by white background. Images were produced with an EntoVision Premium Portable Imaging System, comprising a Leica M16 zoom lens, a JVC KY-75U 3CCD digital camera and a portable computer workstation running EntoVision Imaging Suite software (GT Vision, Hagerstown, MD U.S.A.). Cartograph v5.6.0 (Microvision, Evry, France) software was subsequently used to merge an image series (representing about ten to twenty focal planes), producing a single image with increased depth of field. Illumination was achieved using a “quadrant” setup, with four fibre optic light guides stemming from two individual light "sources (Leica CLS 150 X). Images were edited using Adobe Photoshop CS4 software.

### Data availability

The fig wasp material examined here is held in the collection of JYR and is available for viewing upon request.

## Electronic supplementary material

Supplementary Information
