# Supplementary material for: Pollination of Ficus elastica: India rubber re-establishes sexual reproduction in Singapore
Source: Sci Rep. 2017 Sep 14;7:11616. doi: 10.1038/s41598-017-09873-z (PMC5599632; doi:10.1038/s41598-017-09873-z)
Supplement: Supplementary file 1 — Supplementary Information [file 41598_2017_9873_MOESM1_ESM.pdf]

Pollination of *Ficus elastica*: India rubber re-establishes sexual reproduction in Singapore Supplementary Information

Rhett D. Harrison[1], Kwek Yan Chong[2], Nguyet Minh Pham[2], Alex T.K. Yee[2], Chow Khoon Yeo[2], Hugh T.W. Tan[2], Jean-Yves Rasplus[3]

[1]World Agroforest Centre (ICRAF), East & Southern Africa Regional Office, 13 Elm Road, Woodlands, Lusaka, Zambia

[2]Department of Biological Sciences, National University of Singapore, 14 Science Drive 4, Singapore 117543, Republic of Singapore.

[3]INRA, UMR 1062, 34988 Montferrier-sur-Lez cedex, France

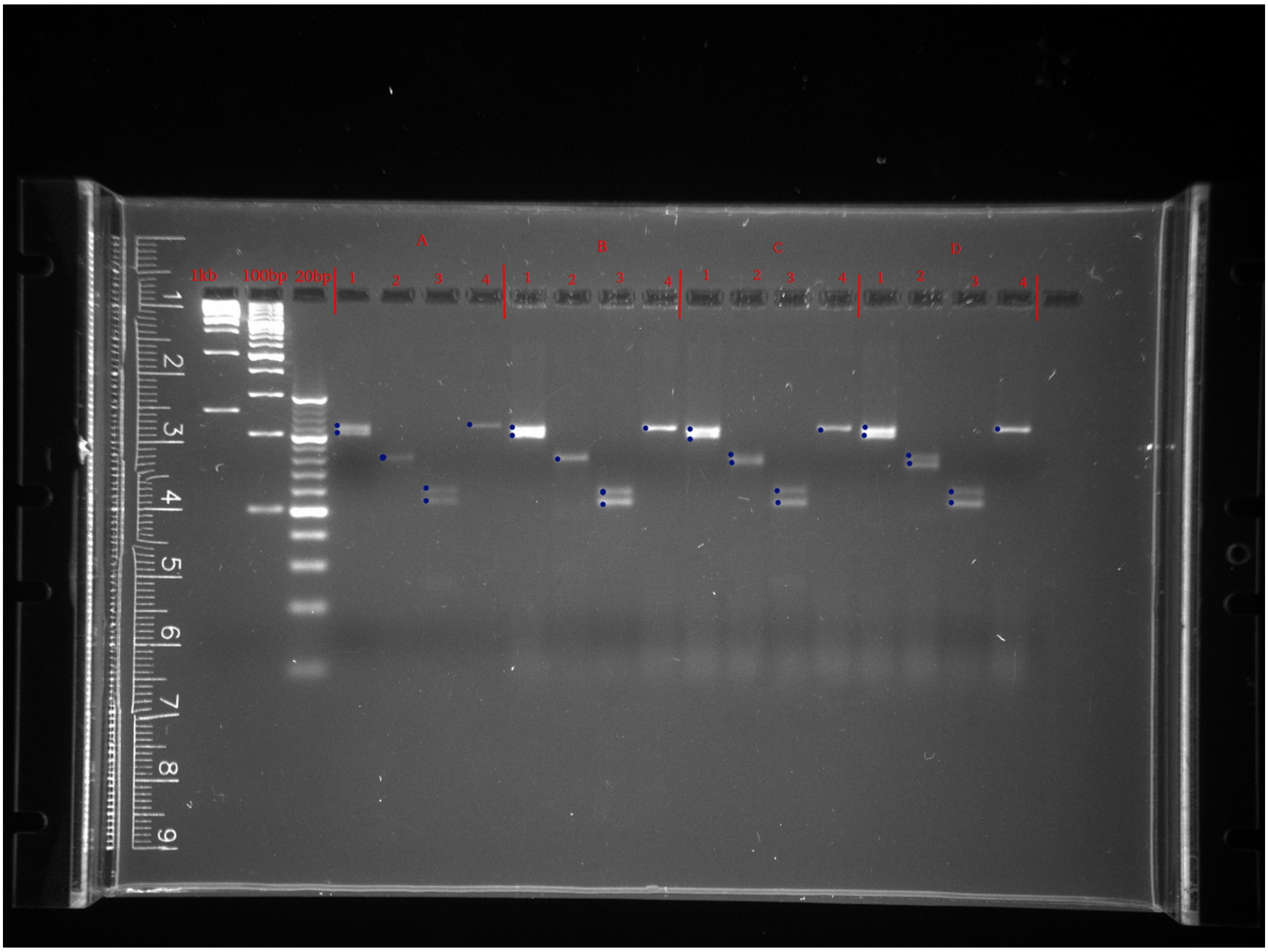

Figure S1 part 1. Results of the microsatellite analyses. A and B are adult trees and C and D were nearby seedlings. All four were found around the Chinese Heritage House on the Nanyang Technological University (NTU) campus. Eight primer pairs were used: MFC1 , MFC2, MFC3, MFC4, MFC5, MFC6, MFC7, MFC8 identified by Khadari et al. (2001). The seedlings are not identical with the adult trees, differing in one of a pair of alleles in MFC2 and MFC5 each and are also not genetically identical (differing in one allele at MFC5).

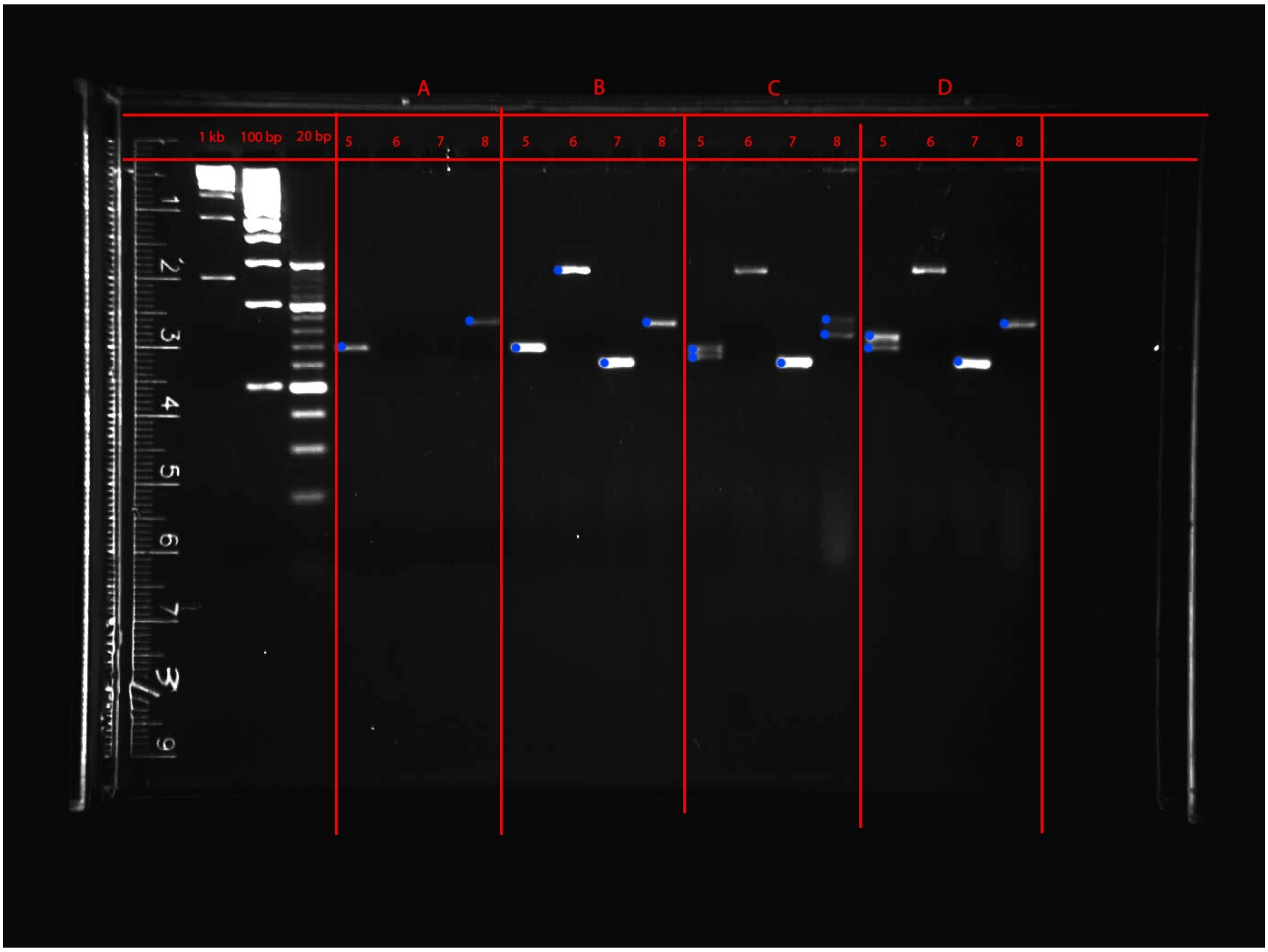

Figure S1 part 2. Results of the microsatellite analyses. A and B are adult trees and C and D were nearby seedlings. All four were found around the Chinese Heritage House on the Nanyang Technological University (NTU) campus. Eight primer pairs were used: MFC1 , MFC2, MFC3, MFC4, MFC5, MFC6, MFC7, MFC8 identified by Khadari et al. (2001). The seedlings are not identical with the adult trees, differing in one of a pair of alleles in MFC2 and MFC5 each and are also not genetically identical (differing in one allele at MFC5).

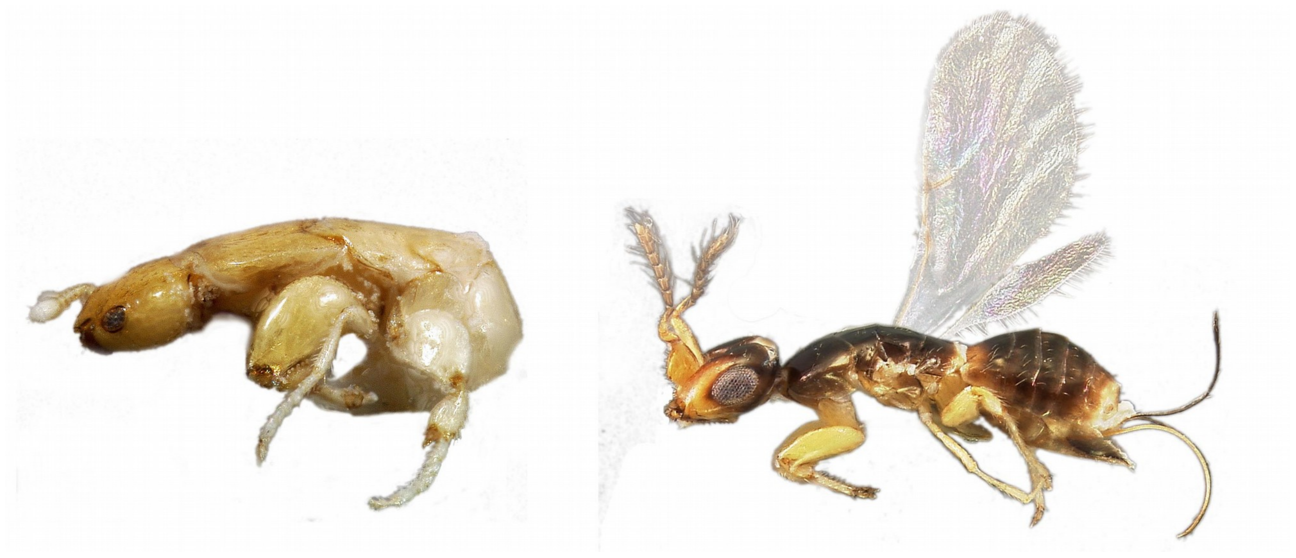

Figure S2. Images of *Platyscapa clavigera* (Mayr 1885) collected in Singapore and imaged using an EntoVision Premium Portable Imaging System, comprising a Leica M16 zoom lens and a JVC KY-75U 3CCD digital camera.
